# Heterogeneity of post-COVID impairment: interim analysis of a prospective study from Czechia

**DOI:** 10.1186/s12985-021-01546-8

**Published:** 2021-04-12

**Authors:** Mikulas Skala, Michal Svoboda, Michal Kopecky, Eva Kocova, Martin Hyrsl, Michal Homolac, Viktor Chrobok, Pavel Bostik, Miroslav Fajfr, Petr Prasil, Stanislav Plisek, Radek Sleha, Vladimir Koblizek

**Affiliations:** 1 Department of Pneumology, University Hospital Hradec Kralove, Hradec Kralove, Czechia; 2 Faculty of Medicine in Hradec Kralove, Charles University, Prague, Czechia; 3 Masaryk University Institute of Biostatistics and Analyses, Brno, Czechia; 4 Department of Radiology, University Hospital Hradec Kralove, Hradec Kralove, Czechia; 5 Department of Otorhinolaryngology and Head and Neck Surgery, University Hospital Hradec Kralove, Hradec Kralove, Czechia; 6 Institute of Medical Microbiology, University Hospital Hradec Kralove, Hradec Kralove, Czechia; 7 Faculty of Military Health Science Hradec Kralove, University of Defense, Brno, Czechia; 8 Department of Infectious Diseases, University Hospital Hradec Kralove, Hradec Kralove, Czechia

**Keywords:** Post-COVID, Symptom, Lung function, 6-min Walk Test, Computer tomography

## Abstract

We stratified post-COVID patients into four newly established clinical groups based on the presence or absence of at least one subjective respiratory symptom and at least one objective sign of pulmonary involvement. Nearly half of outpatients and one third of hospitalized post-COVID patients had objective signs of pulmonary involvement without accompanying subjective respiratory symptoms three months after diagnosis.

By 29nd January 2021, 100 million cases and 2.1 million deaths due to COVID-19 occurred globally [1]. Similar to after the SARS epidemic, a small proportion of patients who recovered from COVID-19 experience sequelae of various severity [2, 3]. Considering the global spread of the disease and the chronic health impact it not seldom imposes, it is meaningful to develop a simple and practicable tool to stratify post-COVID-19 patients.

In spring and summer 2020, the incidence of COVID-19 in the Czech Republic was significantly lower than in some other European countries. The Hradec Kralove District (551.000 inhabitants) had just 182 SARS-CoV-2 PCR-positive patients, of which 24 were hospitalized and three died. All survivors above 18 years of age were offered participation in our one-year prospective observational study. A total of 102 patients were included in the present analysis (i.e. 57% of all positive cases in our district). Of these, 15 were hospitalized (71% of all hospitalized) and 87 treated as outpatients (55% of all outpatients). The first follow-up took place 3 months after the COVID-19 diagnosis. In winter 2020–2021, we continued our prospective data collection to elucidate the real trajectory of patients who recovered from acute COVID-19. Currently, more than 400 patients are followed. We are writing this letter to present the first preliminary results and to introduce a proposal for a novel simple stratification of post-COVID patients to navigate their follow-up care.

We use patients’ demographics and personal history as documented in hospital records. Subjective respiratory and extra-pulmonary symptoms were assessed with a structured physician–patient interview using validated questionnaires assessing respiratory symptoms, anxiety, depression and posttraumatic syndrome. Functional pulmonary examination, 6-min Walk Test (6-MWT) and chest high resolution computer tomography (HRCT) is used to assess objective signs of pulmonary involvement (PI) in all patients. Laboratory results including antibodies against SARS-CoV-2, cellular immunity parameters, C-reactive protein (CRP), and D-dimers are also recorded. HRCTs are assessed by a multidisciplinary committee. Only the HRCT findings that cannot be explained with patient personal history and have not been seen in a control cohort (patients examined with chest HRCT following admission to our hospital Emergency department for suspected pulmonary embolization) are considered as pathological. The control cohort is comparable to the study group with respect to gender, age, BMI, any respiratory comorbidities and nicotine exposure.

A total of 102 patients were included in the study between June and October 2020 and had their first follow-up examination three months from the COVID-19 diagnosis. The mean age was 46.7 years, there were slightly more women (55 women) and the majority of patients (75%) had no comorbidities. The majority of patients were symptomatic at the time they were PCR-positive (n = 99, i.e. 97%). Three months from the onset of the disease, 56 (55%) patients had persistent subjective symptoms they did not have prior to COVID-19. Respiratory symptoms were reported by 35% of patients (23% dyspnoea, 14% cough, 13% pain/chest discomfort), non-pulmonary symptoms mostly included fatigue (22%) and anosmia (21%) more in Table 1. Pulmonary examination most frequently showed reduced pulmonary diffusion (40%). Desaturation during 6-MWT was seen in 11% of patients. HRCTs showed a pathology with causational association to previous COVID-19 in 47% of patients: air trapping (32%), linear opacity (16%), bronchiectasis (12%), multifocal ground glass opacity (11%), fibrosis (6%), and consolidation (2%). Patients with elevated D-dimers underwent CT angiography; none of them had residual pulmonary emboli. In some patients, laboratory results showed persistent inflammatory response (CRP elevation in 11%) and increased pro-coagulation activity (D-dimer elevation in 10%).

**Table 1:** Extra-pulmonary symptoms three months after COVID (N = 102)

Extra-pulmonary symptoms	n (%)
Fatigue	22 (21.6%)
Loss of smell	21 (20.6%)
Loss of taste	7 (6.9%)
Cephalea	6 (5.9%)
Memory impairment	5 (4.9%)
Arthragia/myalgia	4 (3.9%)
Conjunctivitis	2 (2.0%)
Dyspepsia	2 (2.0%)
Subfebrile	1 (1.0%)
Others	14 (13.7%)
At least one non-pulmonary symptom	47 (46.1%)

Table provides an overview of all extra-pulmonary symptoms of patients three months after the diagnosis of COVID-19. Some patients reported more than one symptom

We stratified post-COVID patients into four groups based on the presence or absence of at least one subjective respiratory symptom and at least one objective sign of PI, i.e. reduction in pulmonary diffusion and/or exertion desaturation of oxygen and/or HRCT pathology with suspected post-COVID aetiology. Group A patients do not have any symptoms or signs of PI. Group B patients have symptoms without objective signs of PI. Group C patients have no symptoms but do have objective signs of PI. Group D patients suffer from symptoms and objective signs of PI. Moreover, to enable a comprehensive evaluation of the data, patients with extra-pulmonary symptoms in each group were additionally marked with letter E. The stratification is detailed in the Fig. 1.

**Fig. 1:**
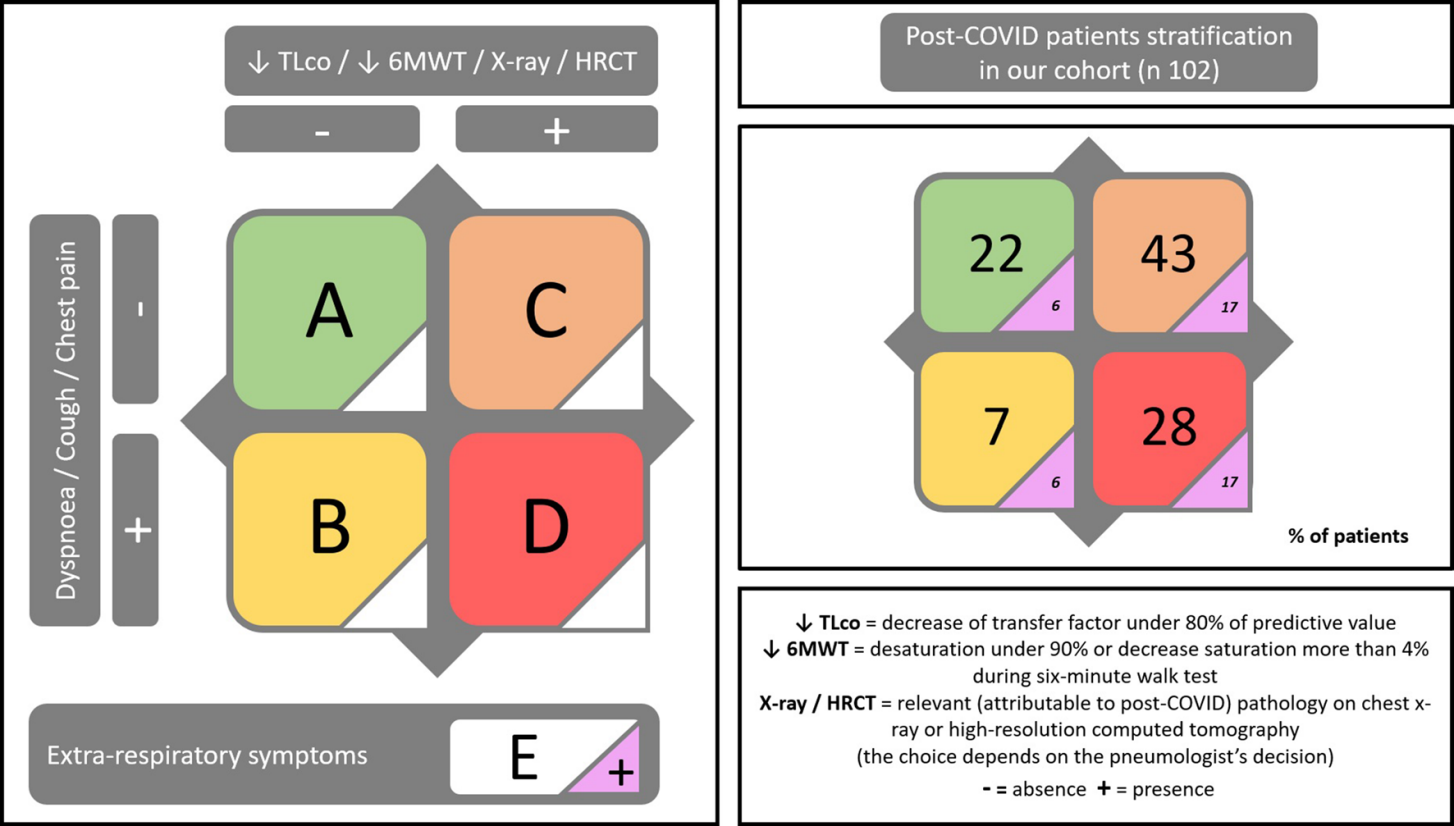
Left side: post-COVID stratification groups. (A) No respiratory symptoms, no objective signs of pulmonary involvement (PI), (B) respiratory symptoms without objective signs of PI, (C) no respiratory symptoms but objective signs of PI, (D) respiratory symptoms and objective signs of PI, (E) extra-pulmonary symptoms (additional mark to group A–D). Example of use—a patient with persistent cough and fatigue without objective signs of PI is classified as B/E. Right side: results of applied use of post-COVID stratification on our study patient cohort

When applied to our study cohort, our post-COVID patient stratification was independent of patient gender, BMI or smoking history, while it correlated with age. In the outpatient cohort (n = 87) three months post diagnosis, 23% of patients were in group A (no subjective respiratory symptoms and no objective signs of PI), 8% in group B (symptoms with no signs of PI), 46% in group C (no symptoms with signs of PI) and 23% in group D (symptoms and signs of PI). Extra-pulmonary symptoms were reported by 43% of outpatient cohort (added E). In hospitalized patients (n = 15), the stratification three months after the diagnosis was as follows: A—13%, B—0%, C—33%, D—54% and 67% with added E (Fig. 1).

Therefore, the majority of previously hospitalized patients had subjective symptoms and objective signs of PI. Nearly half of outpatients and one third of hospitalized patients had objective signs of PI without accompanying subjective symptoms. Furthermore, in our cohort, there is a statistically significant correlation between the presence of respiratory symptoms (CAT, mMRC; groups B and D) and higher level of anxiety (Beck) and depression (Zung). We have observed a clear trend towards more frequent hyper-inflammatory and hyper-coagulation status in Groups C and D.

With the ongoing pandemic, more data on chronic impact of COVID-19 called post-COVID are available. Even though pulmonary tissue is the predominant system involved during the acute phase of COVID-19 and the available data suggest that, in addition to fatigue, respiratory symptoms are the main post-COVID symptoms, the chronic consequences might involve multiple organs [4–6]. In patients hospitalized for COVID-19, respiratory sequelae are common after four months from discharge [7] and patients with more severe disease course during hospitalization have more severe pulmonary injury] [8]. The recently presented data from a large post-COVID cohort from Italy showed fatigue and breathless to be the most common post-COVID symptoms [9]. On the other hand, many patients report physical symptoms without having any lasting physiological injury. [10].

However, other organ systems must not be overlooked and it is likely that other non-pulmonary complications/sequelae of COVID-19 will be seen more frequently. In addition to assessing respiratory symptoms and objective signs of PI, our study will continue to also gather data on extra-pulmonary signs of post-COVID and the extra-pulmonary signs are also included in the proposed stratification.

The National Institute for Health and Care Excellence defines post-COVID syndrome as a group of signs and symptoms that continue for 12 weeks or longer from the disease onset [6]. According to another definition, the post-COVID-19 patients report „lasting effects of the infection “ or they have usual symptoms persisting significantly longer than expected [6, 7, 11]. Various tools to detect post-COVID sequelae are being developed based on patient-reported, subjective, symptoms, e.g. the Post-COVID Functional Status (PCFS) scale [12]. Structural post-COVID PI is seen mainly in hospitalized patients [13]. Some authors newly suggest that the phenomenon of symptomatic post-COVID may not be directly attributable to the post-viral impairment and, instead, these may be biopsychosocial effects of COVID-19 [14].

Our data mainly advert to the patients who have no respiratory symptoms three months post-COVID-19 diagnosis but have objective signs of PI (not explained through their personal history). Therefore, these patients (Group C patients in the proposed stratification) may not fulfil the aforementioned post-COVID definitions focusing on the presence of subjective symptoms. This group can frequently be seen among COVID-19 outpatients.

The small single-centre patient sample size certainly needs to be recognized as a limitation of the present data. It should, nevertheless, be acknowledged that the cohort included more than 50% of all post-COVID patients from the first wave of pandemic in spring 2020. We are also aware that the proposed stratification tool might be more suitable for healthcare systems with wide availability of pulmonary diffusion measurement in the care of post-COVID patients. Our data are also limited by the lack of baseline pre-COVID HRCTs in outpatients with mild course of the disease. Moreover, in the absence of sufficient data, it is uncertain, whether the PI we observed can be considered as a COVID-19-specific viral pneumonia in terms of long-term radiological changes and their clinical relevance [15]. Further follow-up in these patients is required to determine whether the observed changes are reversible.

The clinical relevance of our proposed post-COVID patient clinical stratification tool must, understandably, be validated and possibly corrected using data from other centres. This correspondence is an invitation to such a joint activity.

## Conclusion

Considering the continuation of the global COVID-19 pandemic and the growing data on its chronic impact, there is a clear need for a simple clinical tool to triage post-COVID patients and organize follow-up medical care. We established a new tool for rapid stratification of post-COVID patients based on a targeted search for respiratory and extra-pulmonary symptoms and assessment of objective signs of pulmonary involvement. When applied to our patient cohort comprising 57% of all positive „spring 2020 cases “, this stratification identified a high-risk group of patients with post-COVID syndrome and objective pulmonary involvement (group D), a group with post-COVID syndrome but without objective pathology (group B) and, importantly, an asymptomatic group with pulmonary involvement (group C). In our opinion, this group (C) warrants increased attention. Even though at present, these patients have no respiratory symptoms, the exertional respiratory insufficiency and/or reduced pulmonary diffusion and/or some of the CT/X-ray findings have a potential to develop into symptomatic progression and permanent damage. This is supported by the clear trend towards more frequent hyper-inflammatory and hyper-coagulation status we observed in these patients (similarly to group D). However, since this group is not meeting the current criteria for post-COVID syndrome based on symptoms only, it might be slipping the attention of health care providers. In addition, our stratification identifies patients requiring care of medical specialities other than pneumologists (the added E group).

## Data Availability

The datasets used and/or analysed during the current study are available from the corresponding author on a reasonable request.

## Abbreviations

6-MWT: 6-Min Walk Test
CAT: COPD Assessment Test
CRP: C-reactive protein
HRCT: High resolution computer tomography
mMRC: Modified Medical Research Dyspnoea Scale
PCFS: Post-COVID functional status
PI: Pulmonary involvement
TLco: Transfer factor for carbon monoxide or diffusing capacity for carbon monoxide
